# Evaluation of Wood Composite Sandwich Panels as a Promising Renewable Building Material

**DOI:** 10.3390/ma14082083

**Published:** 2021-04-20

**Authors:** Mostafa Mohammadabadi, Vikram Yadama, James Daniel Dolan

**Affiliations:** 1 Materials Science and Engineering Program and Composite Materials and Engineering Center, Washington State University, Pullman, WA 99164, USA; m.mohammadabadi@wsu.edu; 2 Department of Civil and Environmental Engineering and Composite Materials and Engineering Center, Washington State University, Pullman, WA 99164, USA; jddolan@wsu.edu

**Keywords:** wood composites, sandwich panel, building material, bending stiffness, R-value, axial compression, biaxial corrugated core

## Abstract

During this study, full-size wood composite sandwich panels, 1.2 m by 2.4 m (4 ft by 8 ft), with a biaxial corrugated core were evaluated as a building construction material. Considering the applications of this new building material, including roof, floor, and wall paneling, sandwich panels with one and two corrugated core(s) were fabricated and experimentally evaluated. Since primary loads applied on these sandwich panels during their service life are live load, snow load, wind, and gravity loads, their bending and compression behavior were investigated. To improve the thermal characteristics, the cavities within the sandwich panels created by the corrugated geometry of the core were filled with a closed-cell foam. The R-values of the sandwich panels were measured to evaluate their energy performance. Comparison of the weight indicated that fabrication of a corrugated panel needs 74% less strands and, as a result, less resin compared to a strand-based composite panel, such as oriented strand board (OSB), of the same size and same density. Bending results revealed that one-layer core sandwich panels with floor applications under a 4.79 kPa (100 psf) bending load are able to meet the smallest deflection limit of *L*/360 when the span length (*L*) is 137.16 cm (54 in) or less. The ultimate capacity of two-layered core sandwich panels as a wall member was 94% and 158% higher than the traditional walls with studs under bending and axial compressive loads, respectively. Two-layered core sandwich panels also showed a higher ultimate capacity compared to structural insulated panels (SIP), at 470% and 235% more in bending and axial compression, respectively. Furthermore, normalized R-values, the thermal resistance, of these sandwich panels, even with the presence of thermal bridging due to the core geometry, was about 114% and 109% higher than plywood and oriented strand board, respectively.

## 1. Introduction

Considering the good properties and superior advantages of natural fibers over synthetic fibers, such as low weight, low cost, less damage to processing equipment, being abundant, safe to handle, and a minimal health hazard [1], natural fiber composites have begun to penetrate many markets including the aerospace [2,3], automotive [4,5], medical [6,7], electronic [8], and food [9] industries. Natural fiber production, for instance, on average uses 60% less energy than glass fiber production, and results in a lower cost and lower air emissions [10]. Considering sustainability and CO_2_ emission in addition to the advantages of natural fibers over synthetic ones, bio-based composite materials that are environmentally friendly, renewable, and biodegradable have attracted the attention of many researchers. However, development in Engineered Wood Products (EWP) is not noticeable as recent advancements in wood products are summarized as industrial chemicals, biofuels, and energy from woody materials, and as use of cellulose nanomaterials in the development of different products such as cement-based material additives, food coatings, and transparent flexible electronics [11,12]. EWPs such as laminated veneer lumber (LVL), plywood, oriented strand board (OSB), and mass timber products such as glue-laminated timber (Glulam), cross laminated timber (CLT), and structural composite lumber (SCL) that are used in building construction have been around for decades, for instance. Since the application of mass timber products in the construction of tall buildings is of interest to the industry and the research community these days, several studies have evaluated different aspects of these products [13,14,15]. Additionally, limited work has been conducted on developing wood-based composite sandwich panels with three-dimensional cores for use in building envelopes. Therefore, further research to develop new EWPs with a higher load capacity, especially out of small diameter trees, can open new business opportunities and also address both CO_2_ emission and sustainability through the efficient use of natural resources.

Sandwich structures that consist of two face-sheets and a core layer were originally developed and used in the aerospace industry due to their high performance and stiffness-to-weight ratio and have received a lot of attention from researchers [16,17,18]. The core can have a continuous geometry, such as metallic foam, or a discrete with periodic geometry, such as honeycomb and corrugated cores. Sandwich structures with hollow cores not only have a higher stiffness-to-weight ratio compared to continuous ones, but the cavities created by the discrete geometry of the core also can be used to increase the thermal and acoustic performance of the sandwich panel [19]. Owning to these merits, sandwich structures are not limited only to aerospace and also can be found in automotive, marine, and civil industries. This concept also has been borrowed by the building industry to develop construction materials such as the structural insulated panel (SIP) that is composed of an insulated foam core between OSB face-sheets. However, drawbacks of SIPs are that expanded polystyrene foam cores can easily burn and are not environmentally preferred materials; the walls are not breathable and, thus, the buildings require a mechanical ventilation system to control moisture; they do not offer a high load-carrying capacity, so strengthening and retrofitting against axial, flexural, and lateral loads are required [20]. To tackle these drawbacks, and to develop lightweight and high-performance products, researchers have recently adopted discrete core geometry in the fabrication of EWPs.

Using a wet process, Hunt and Winandy [21] fabricated a 3-D engineered fiberboard core sandwich panel with a uniaxial corrugated core geometry. A hollow core was developed using roll forming of plywood by heated rolls and was bonded to 3-ply veneer [19,22]. Using a matched-die molding process, Way et al. [23] produced a molded core made of wood strands bonded to OSB face-sheets. A corrugated cardboard core was bonded to flax fiber composite face-sheets to fabricate multilayered sandwich panels [24]. To fabricate a sandwich panel from beech wood, two corrugated panel were bonded together, then ripped multiple times to produce a single oval-shaped core cell [25]. Then, core cells were sandwiched between formed veneers to make a block that was sawn into slices and used as the core of the sandwich panel. Thin-walled sandwich panels were developed using wood strands for the corrugated core as well as for the flat face plies [26,27,28]. Regarding all cases, these sandwich panels with discrete cores have been manufactured and investigated in lab-scale experiments. Since all behavior and deformations of a structural member cannot be captured and predicted based on the results of a lab-scale experiment, it is necessary to fabricate and evaluate larger sandwich panels to evaluate their performance.

**Figure 1:**
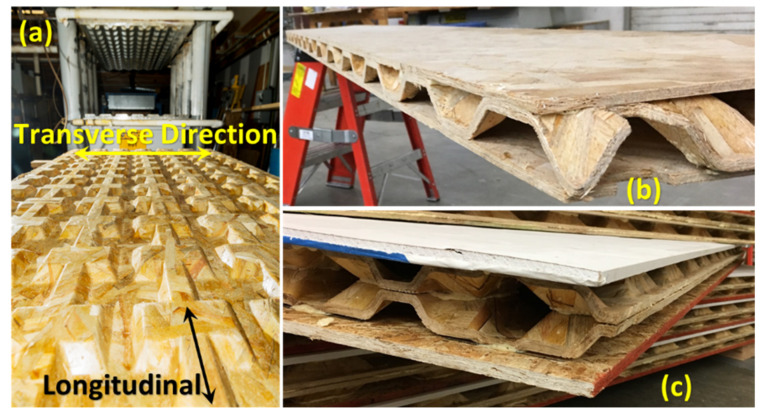
(**a**) Full size biaxial corrugated panel used as a core to fabricate (**b**) one-layered (**c**) two-layered core sandwich panels.

Using a finite element (FE) model validated for a biaxial corrugated geometry [29], a new geometry was designed to be used as a core in the fabrication of multilayered sandwich panels [30]. The objective of this study is to fabricate full-size (1.2 m by 2.4 m) wood strand composite sandwich panels with cores having this new biaxial corrugated configuration (Figure 1a) and evaluate their structural and thermal performance. Sandwich panels with a one-layered core (Figure 1b) were manufactured for roofing and flooring applications, and those with a two-layered core (Figure 1c) for a wall element. Considering the primary loads applied during their service life, sandwich panels were subjected to bending and/or axial compression testing. To improve the thermal performance, the cavities created by the corrugated geometry of the core were filled with insulating foam, and the thermal conductivity was measured.

Only the strength and deflection performances of the new panel concept were considered in this study. Other performance criteria, such as vibration, sound transmission, etc., were beyond the scope of this study.

This paper covers the fabrication of a wood strand composite panel with a biaxial corrugated geometry used as a core to make multilayered sandwich panels as a building material, the mechanical testing procedure to evaluate their performance under applied loads during their service life, the procedures used to quantify the thermal resistance properties, and the test results. Conclusions are then drawn based on these results and comparisons made with some available wood-based products on the market.

## 2. Panel Fabrication

**Figure 2:**
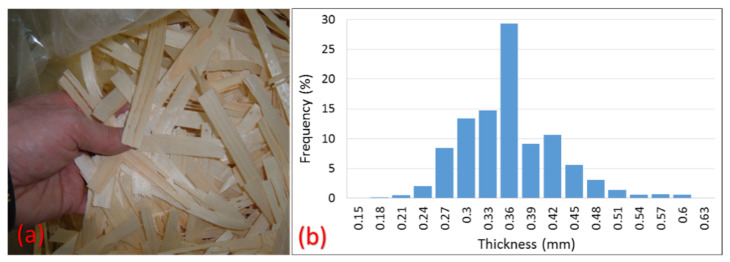
(**a**) Wood strands and (**b**) frequency distribution graph of their thickness.

Low-quality spruce-pine-fir lumber provided by the Idaho Forest Group were used to produce thin wood strands. A laboratory disk strander (CAE double blade mounted disk) operating at a rotational speed of 500 rpm was set to strand the lumber into 154 mm × 19 mm × 0.36 mm (nominal dimensions of *L* × *W* × *t*) strands, as shown in Figure 2. Even though special care and effort was taken to target the strand thickness of 0.36 mm, it was difficult to avoid thickness variation. Measurement for more than 1000 strands showed an average thickness of 0.34 mm and a 2.6% coefficient of variation (COV). The percentage frequency distribution of the thickness of the strands is shown in Figure 2. After drying strands to a target moisture content of 3–5%, they were mixed with 8% phenol formaldehyde resin in a drum blender.

**Figure 3:**
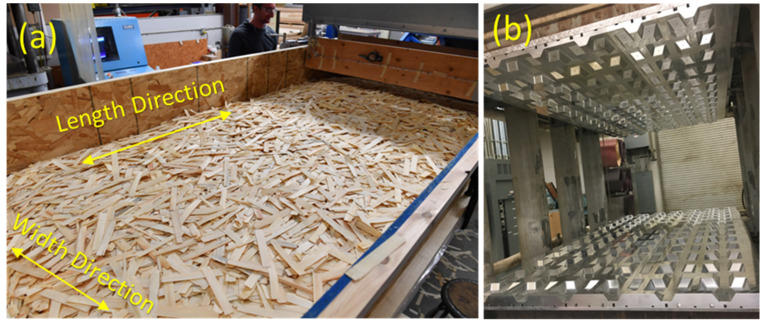
(**a**) Wood-strand mat (*preform*), and (**b**) Aluminum matched-die mold.

Using a conveyor belt system, strands sprayed with resin were carried to an orientor to be aligned ±30° with respect to the direction that is parallel to the length of the panels or the longitudinal direction. Length or longitudinal and width or transverse directions of the preform and, subsequently, of the finished panel are shown in Figure 1a and Figure 3a. A wood strand mat or preform, shown in Figure 3a, was built by the accumulation of unidirectionally aligned strands. Using a matched-die mold shown in Figure 3b, the *preform* was consolidated and hot-pressed for 6 min at an operating temperature of 160 °C to fabricate a biaxial corrugated panel, as shown in Figure 1a. The geometry of the core can be defined by describing the configuration of one-unit cell (UC) that is a repeating element along the length and width of the corrugated panel [31]. The same process, without using the mold, was adopted to manufacture 6.35 mm thick flat panels with wood strands.

**Figure 4:**
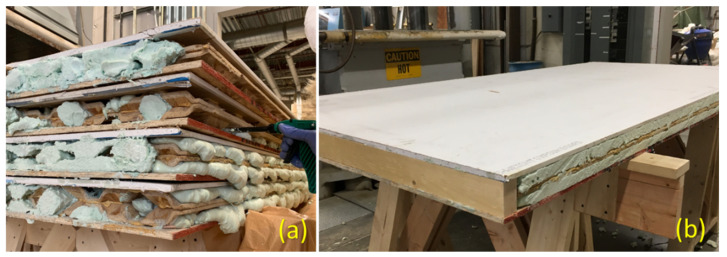
(**a**) Filling two-layered core sandwich panels with spray foam (**b**) trimmed foam and wall prepared to use.

To fabricate the sandwich panels, all components, flat layers, and corrugated core(s), were bonded using a polyurethane adhesive (LOCTITE HB X452 PURBOND, Henkel) at room temperature. Regarding roof and floor applications, one-layered core sandwich panels, as shown in Figure 1b, were fabricated by bonding wood-strand flat panels to each side of a corrugated layer. However, to increase the in-plane load-carrying capacity and insulation capacity of panels used as a wall member, two corrugated panels were used in the core, as shown in Figure 1c. To be consistent with traditionally constructed walls with studs, 10.5 mm thick OSB and 12.7 mm thick drywall were used as face-sheets for the two-layered core sandwich panels. To increase the energy performance of the wall members, the cavities of two-layered core sandwich panels were filled with 2-part closed-cell foam by spraying the foam directly into the cavities (Figure 4a). After trimming the excess foam along the edges, lumber was inserted on each end of the wall panels between face plies to act as the top and bottom plates (Figure 4b).

## 3. Experimental Evaluation

**Figure 5:**
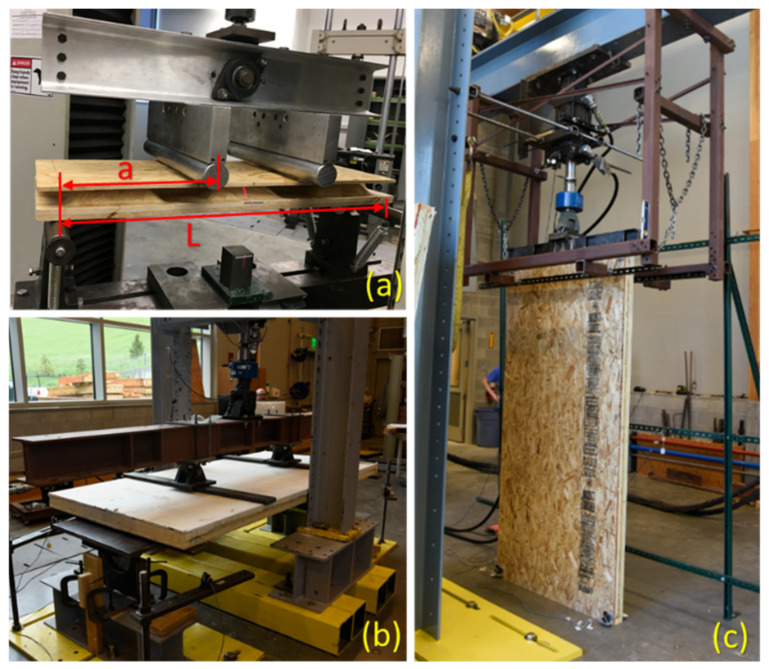
Experimental testing; (**a**) small scale bending of 559 mm long and 1-UC wide specimen from a one-layered core, large scale (**b**) bending, and (**c**) compression testing of a commercial-size two-layered core sandwich panel.

Roof and floor panels must be designed to support a bending load generated by snow, as well as live, dead, and wind loads. To evaluate the performance of commercial-size (full-size) roof and floor panels for deflection and ultimate capacity under a bending load, ASTM E2322 [32] and ASTM E72 [33], which define a loading span as half of the span length (*L*/2), are recommended. Based on the preliminary testing, failure of the face-sheets due to tension or compression was the most common failure in specimens under a bending load, and such a failure was more likely than the core’s failure since both face-sheets and core were manufactured from the same materials—wood strands. Therefore, following ASTM D7249 [34], which has the same test configuration and four-point bending as ASTM E2322 [32] and ASTM E72 [33], but covers the determination of face ply properties of flat sandwich constructions under a bending load, was preferred. Additionally, ASTM D7249 [34] offers two different loading spans, one-third and one-half of the span length (*L*/3 and *L*/2), where the smallest was selected to conduct experimental testing, while both ASTM E2322 [32] and ASTM E72 [33] define a loading span as half of the span length (*L*/2). Since a smaller loading span results in a higher maximum bending moment and an increase in the normal stress on the face-sheets, it helps to reveal the actual performance of the face-sheets and, therefore, that of the sandwich structures with one corrugated core under a bending load. Therefore, specimens with different span lengths, cut from a one-layered core sandwich panel, were subjected to a four-point bending test where the loading span was one-third of the span length. The bending test of a specimen with the smallest span length (559 mm) is shown in Figure 5a. Wall members, however, during their service life are subjected to both bending and axial compression due to wind and gravity loads, respectively. Therefore, following ASTM E72 [33], commercial-size two-layered core sandwich panels as a wall member were evaluated using bending and compression testing, as shown in Figure 5b,c. The dimensions and details of all bending and compression specimens are given in Table 1. The bending stiffness of specimens submitted to a four-point flexural test can be calculated as:

(1) $$EI=\frac{Pa}{48\Delta }\left(3L^{2}-4a^{2}\right)$$

where, *P*, Δ, and *L* are the bending load, deflection at mid-span, and span length, respectively. The constant “*a*” is the distance between the support and loading point which is shown in Figure 5a.

**Figure 6:**
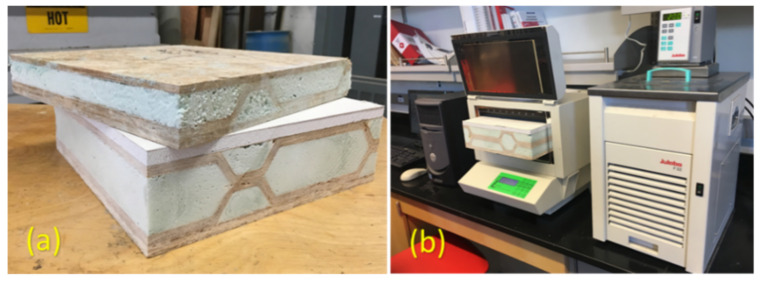
Thermal test (**a**) samples of both sandwich panels (**b**) heat flow meter.

Thermal resistance, R, is a measure of resistance to heat flow and can be calculated by taking the reciprocal of the thermal conductivity, and is a common property used to determine how well a material insulates. To determine thermal conductivity, both one- and two-layered core sandwich panels were filled with closed-cell polyurethane foam (Foam it Green), and then cut into 305 mm by 305 mm specimens, as shown in Figure 6a. A Fox 304™ heat flow meter (LaserComp, Saugus, MA, USA) shown in Figure 6b was used to determine the thermal conductivity based on the Fourier Law, as per ASTM C1045 [35]. Since thermal conductivity is temperature-dependent, different temperatures were used to measure the thermal conductivity. However, a mean temperature of 24 °C (75 K) should be used to evaluate thermal properties of the building materials [36,37].

## 4. Results and Discussions

After fabrication of flat and corrugated panels, twenty-two density measurements taken from a variety of locations across both panels indicated an average density of 800 kg/m^3^ (50 pcf), a specific gravity of 0.8, with a coefficient of variation of 15.3% for the wood strand composite. The target length, width, wall thickness, and height for the corrugated core were 2438 mm (8 ft.), 1219 mm (4 ft.), 7.6 mm (0.3 in), and 35 mm (1.375 in), respectively [38]. When the weight of the corrugated panel was compared to that of an OSB panel of the same size and same density (809 kg/m^3^), the corrugated panel was 3.86 times lighter than the OSB panel. Fabrication of corrugated panels required 74% less supplies (strand and, as a result, resin as well) compared to an OSB of the same size and same density, in other words.

Considering the application of the sandwich panels, results are divided into three parts. The first one reports the data of one-layered core sandwich panels for roof/floor applications, while the second part presents the test results of two-layered core sandwich panels when used as a wall member. The results of the thermal conductivity test are given in the last subsection.

### 4.1. One-Layered Core Sandwich Panels with Roof/Floor Applications

**Figure 7:**
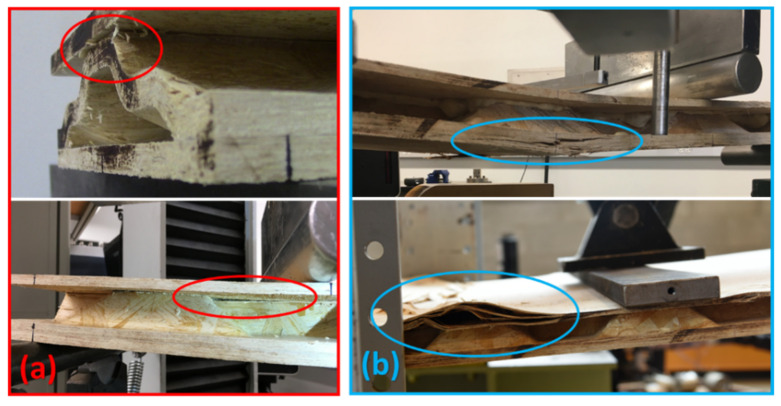
Failure mode of one-layered core sandwich panel (**a**) interfacial debonding (**b**) tension or compression in face-sheet.

When subjected to a four-point bending load, the failure mode of short span specimens including 559 mm and 1092 mm was debonding between the core and the outer layer due to interfacial shear, as shown in Figure 7a. However, long span specimens (1219 mm and 2438 mm) failed due to tension or compression in the face-sheet, as shown in Figure 7b.

**Figure 8:**
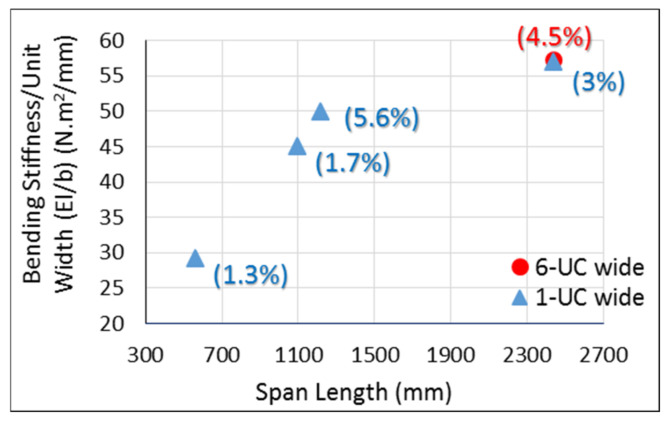
Bending stiffness per unit width for one-layered core sandwich panel (values in parenthesis are COV).

The effective bending stiffness per unit width for one-layered core sandwich panel specimens with different span lengths was computed using Equation (1) and is shown in Figure 8. By increasing the span length, the bending stiffness increases since the effect of shear deformation is reduced. Therefore, the bending stiffness of specimens with a span length of 2438 mm should be considered the effective bending stiffness for sandwich panels with one layer of corrugated core since this span is the typical maximum span for most building applications. However, changing the width had a negligible effect on the bending stiffness of this sandwich panel. Shown in Figure 8, the bending stiffness per unit width of specimens with different widths (1-UC and 6-UC), but the same span length of 2438 mm, did not vary significantly (~1% difference). The wood handbook [39] reports the longitudinal Young’s modulus of 4.41 GPa for OSB panels with an specific gravity (S.G.) of 0.8 (density of 800 kg/m^3^), which can be used to calculate its bending stiffness. Comparison revealed that the bending stiffness of a sandwich panel is 44% higher than an OSB of the same thickness. It should be noted that 800 kg/m^3^ was the density of the core wall and outer layers of the sandwich panel, while the density of a one-layered core sandwich panel was 320 kg/m^3^ (20 pcf), with an S.G. of 0.32. Normalizing the bending stiffness of each panel by its density showed that the result for a one-layered core sandwich panel is 265% higher than that of an OSB. Considering both weight and stiffness, it confirms that this wood-strand-based sandwich panel in a flooring and roofing application shows a higher performance than an OSB as a structural panel.

The modulus of rupture (MOR) is proportional to the maximum moment, which is equal to *M_max_ = F_max_L/*6. Considering the bending results of the specimens with different span lengths, an average maximum moment was computed for the one-layer sandwich panel. The result revealed that the one-layer sandwich panel specimens under a four-point bending test failed when the maximum bending moment in the specimen divided by its width ($M_{max}^{b}$) reached 6.04 N.m/mm.

Structural panels, when used as roof or floor panels, must satisfy a deflection limit through their design process. Considering various loadings and types of applications, this limit can be 1/120, 1/180, 1/240, or 1/360 of the span length of the panel [40], depending on the finish materials attached. To satisfy the design, deflection of the panel for a specific loading and application must not exceed the corresponding limit. Considering the true bending stiffness (*EI*) of the single core sandwich panel, based on Figure 8, required distributed load of q that results in a mid-span deflection of δ in a panel with span length of *L* can be expressed as:

(2) $$q=\frac{384EI\delta }{5L^{4}}\;\;\;\;\;\;\;\;\;\;\;where\;\;\;\;\;\;\;\delta =\frac{L}{120},\;\frac{L}{180},\;\frac{L}{240},\;\frac{L}{360}$$

**Figure 9:**
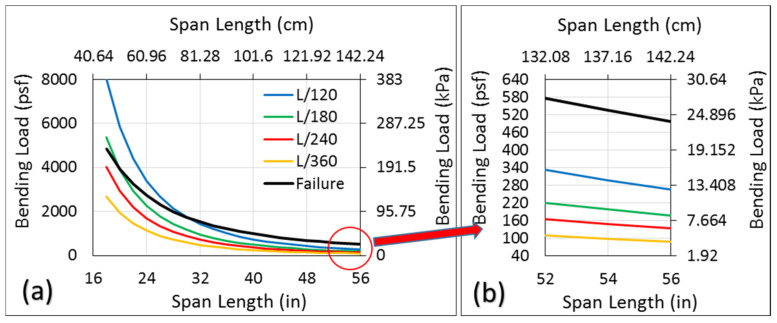
(**a**) Maximum bending load for different span lengths without passing the deflection limits (**b**) a blow-up of the circle area in part a.

The maximum distributed loads that can be applied to a panel corresponding to different deflection limits can be computed using Equation (2) and are plotted in Figure 9a. Regarding all deflection limits, the maximum bending load increases while the span length decreases. Notably, the maximum bending load obtained from the deflection limit (Equation (2)) should not be counted as the load capacity of the specimen, and the failure of the specimen under this load must be checked. To this aim, the distributed load that results in a maximum bending moment of the sandwich panel is computed as follows:

(3) $$w_{max}=\frac{8M_{max}^{b}}{L^{2}}$$

where, $w_{max}$ and *L* are the distributed load at the failure and span length of the specimen, and $M_{max}^{b}$ is the bending moment capacity normalized by the width, which is 6.04 N.m/mm for a one-layered core sandwich panel. The maximum distributed load obtained from Equation (3) for different span lengths also is plotted as a failure curve in Figure 9. Therefore, the maximum distributed bending load that can be applied on the sandwich panel with a single core at a given span length would be the smallest value of the bending load either obtained from the deflection limit (Equation (2)) or the failure curves (Equation (3)). Regarding specimens with a 61 cm (24 in) span length, for instance, the maximum distributed bending load based on a deflection limit of *L*/120, which was 162.14 kPa (3386 psf), is not acceptable since the specimen will fail at a smaller load, which was 130 kPa (2716 psf) as obtained from the failure curve. Notably, for specimens with a span length of 76.2 cm (30 in) and larger, the bending load obtained from any deflection limit can be accepted as the load carrying capacity of the specimen since it falls under the failure curve and, thus, is smaller than the load obtained from the bending moment capacity. A blow-up of Figure 9a, where the load based on deflection limits falls under the failure curve, is presented in Figure 9b. A specimen with a span length of 137.16 cm (54 in) can meet all deflection limits under a distributed bending load of 4.79 kPa (100 psf). It means that if a one-layered core sandwich panel evaluated in this study is used as a floor or a roof panel, the joist span for such a load can be increased to 137.16 cm (54 in) no matter what the deflection limit.

### 4.2. Two-Layered Core Sandwich Panels for Wall Application

**Figure 10:**
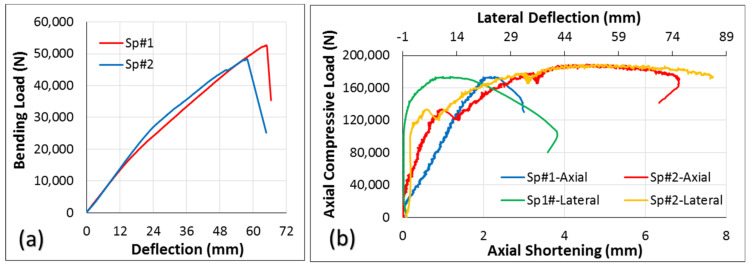
Load-deflection curves for two-layered core sandwich panels under (**a**) bending and (**b**) axial compression tests.

**Figure 11:**
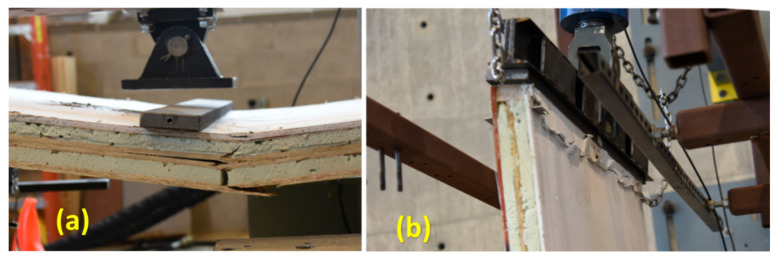
Typical failure of two-layered core sandwich panel under (**a**) bending: tension and (**b**) compression: crushing.

Two sandwich panels (with two layers of the 3-D core) filled with 2-part closed-cell foam were tested in bending, and two in axial compression, studies. Regarding the bending test, load was applied on the inside surface (gypsum) rather than the outside surface (OSB). One reason for this was to understand the effect of suction on the wall caused by wind load, which puts the inside surface in compression. Another reason was to find the actual strength of this two-layered sandwich panel under a bending load, since gypsum is not strong in tension, and an applied load on the outside surface (OSB), which puts the inside surface (gypsum) in tension, possibly resulting in early gypsum failure. Regarding the case of axial compression, both axial shortening and lateral deflection at the mid-height of the wall were collected using displacement transducers (10 in string potentiometers). The load-deflection curves of the two specimens of this sandwich panel configuration under bending and compressive loads are presented in Figure 10. Regarding axial compression, it seems that the panels behaved differently in terms of axial shortening and lateral deflection. However, it should be emphasized that these differences compared to the dimensions of the wall members are negligible. Axial shortening, for instance, differs by about 4 mm while the length of these walls is 2438 mm. The typical failure of the sandwich panel under four-point bending and axial compression tests are shown in Figure 11. Failure under bending was tension failure in the face-sheet, as shown in Figure 11a. Regarding the compression test, since the load was applied along a line located at one-third of the thickness of the specimen from the inside face-sheet (drywall), and not at the center of the wall as per ASTM E72 guidelines [33], failure was due to crushing in the drywall, as shown in Figure 11b.

Bending and compression results of the sandwich panels were compared with those of structural insulated panels (SIP) and traditional stud walls from the literature, in Table 2. Abbasi [41] performed bending and axial compression tests on both the SIP and stud walls that were 165 mm (6.5 in) in thickness, which is thicker than the thickness of the two-layered core sandwich panel (93 mm) in this study. While the OSB was used as a face-sheet on both sides of the SIP, face-sheets for the stud wall were the same as the two-layered core sandwich panel in this study, an OSB on one side and drywall on the other.

Maximum loads from bending and compression were used to find the equivalent distributed load (w). Regarding the compression test, the maximum load was divided by the area of the surface where the force was applied. However, for the bending test, the maximum moment, which occurs at the mid-span due to line load (*P*) or distributed load (w), was the criteria to find the equivalent distributed load as follows:

(4) $$\left.\begin{array}{l} Line\;load\;\left(This\;study\right):\;\;M_{max,\;Line}=\frac{PL}{4}\\ Distributed\;load:\;\;M_{max,\;Dis}=\frac{wL^{2}}{8} \end{array}\right\}M_{max,\;Line}=M_{max,\;Dis}\;\;\to \;\;\;w=\frac{2P}{L}$$

Comparison of the distributed load revealed that the two-layered core sandwich panel was able to carry a 235% and 158% larger distributed axial compression load than the SIP and the stud wall, respectively. The distributed bending load for the sandwich panel was 106% larger than the SIP, while it was 21% lower than that of the stud wall. However, it should be noted again that the SIP and stud walls had different thicknesses and span lengths than the sandwich panel, as given in Table 2. An equivalent distributed load took the span length into account but did not account for a change in the thickness of the panels. Therefore, since the maximum stress (MOR) caused by the line load, which is computed by σ=MC/I=PLC/4I that takes both the thickness and span length into account, is presented in the last column of Table 2, and was used for a fair comparison.

Comparison of the strength revealed that the MOR of the sandwich panel was 470% and 94% higher than that of the SIP and stud walls, respectively. Results of these experiments confirm that two-layered core sandwich panels with the same thickness and span length of SIPs and stud walls would have a higher performance under bending and axial compression loads. The value of COV, as a percentage, is given in parenthesis.

### 4.3. Thermal Performance

Thermal conductivity values estimated for both one- and two-layered core sandwich panels with closed-cell foam in the interior cavities are presented in this subsection. To explore the effect of temperature on the thermal performance of the sandwich panels, specimens were evaluated at different temperatures, and thermal conductivities normalized by thickness are given in Table 3. Comparison of the results shows that the thermal conductivity of both sandwich panels increased when the mean temperature also was increased. Changing the mean temperature from 2.5 °C to 62.5 °C results in a 23% and 20% increase in the thermal conductivity of one- and two-layered core sandwich panels, respectively. Therefore, these sandwich panels have a better energy performance at lower temperatures. Also, for all the mean temperatures, one-layered core specimens showed a lower thermal conductivity than a two-layered one. This difference, which varies from 2.3% to 5.1%, is shown in the last column of Table 3.

**Figure 12:**
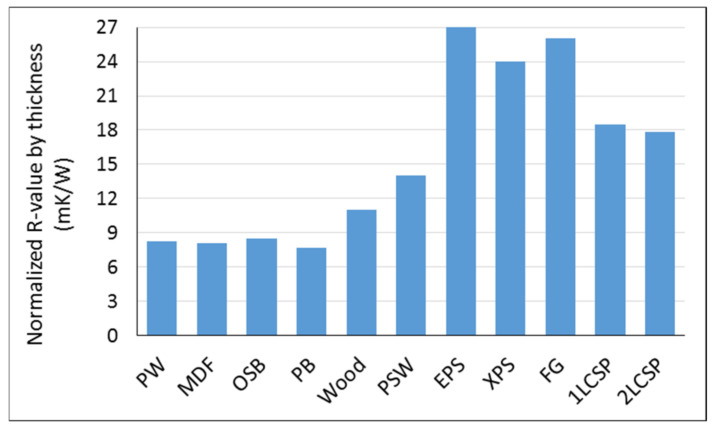
Comparison between normalized R-value of sandwich panel with corrugated core to those of commercially available wood-based and insulator products provided by Kawasaki and Kawai [42].

The R-value of one- and two-layered core sandwich panels (1LCSP and 2LCSP) normalized by thickness is compared in Figure 12 to those of commercially produced wood-based panels, solid wood (Japanese cedar), and commercially available insulators provided by Kawasaki and Kawai [42]. The commercial wood-based products were plywood (PW), medium density fiberboard (MDF), oriented strand board (OSB), particleboard (PB), and plywood-faced sandwich panel with a low-density fiberboard core (PSW), while the commercial insulators were expanded polystyrene foam (EPS), extruded polystyrene foam (XPS), and fiberglass wool (FG).

A comparison of normalized R-values in Figure 12 shows that corrugated core sandwich panels have a higher energy performance, about 120% on average, than commercial wood-based products including PW, MDF, OSB, and PB. Compared to solid wood, the normalized R-value of the sandwich panel is about 65% higher. Sandwich panels indicated a 30% higher normalized R-value than PSW, which was specifically developed as a wood-based insulation material [42]. However, compared to commercial insulators such as EPS, XPS, and FG, the wood strand sandwich panels evaluated in this study presented a lower normalized R-value, at about 30% on average. An advantage of the sandwich panel developed in this study is the versatility to include other materials within the panel construction to further improve the panel’s insulation capacity, as long as the new material does not significantly reduce the required load-carrying capacity.

## 5. Conclusions

Sandwich panels with corrugated core(s) were fabricated as a renewable building construction material. Sandwich panels with one core were introduced for floor and roof applications while, for wall members, two corrugated layers were used as a core. Considering the primary loading applied to these sandwich panels during their service life, bending and compression testing were performed to evaluate their performance. Also, the thermal conductivity of specimens filled with closed-cell foam was measured.

Based on the bending results of specimens at different span lengths, the stiffness and strength of the one-layered core sandwich panel was computed. The load-carrying capacity for different spans was determined based on satisfying the deflection limits and strength of materials. Results revealed that the joist span can be increased to 137.16 cm (54 in) for a 4.79 kPa (100 psf) bending load. A two-layered core sandwich panel as a wall member showed a significantly better performance than some current building products, namely SIP panels and traditional stud walls. The normalized R-value of these sandwich panels was about 120% higher than commercial wood-based products. However, commercial insulators such as EPS, XPS, and FG showed about a 30% higher performance than the sandwich panel products. Results of this study indicate that high-performance wood strand sandwich panel products for flooring, roofing, and panelized walls can be manufactured using small diameter timber from thinning operations to reduce hazardous fuel loads.

The findings of this research indicate that the structural and thermal performances of the 3-D sandwich panel configuration developed for this research provides a significantly improved performance for structural and thermal performances when compared to available wood-based products currently used for wall and floor configurations on the market. Therefore, it is conceivable to prefabricate these types of sandwich panels as panelized building envelope elements to be shipped to the job site for quick construction of a residential home building envelope. This can potentially save building time and labor costs. Additionally, the use of small diameter timber for manufacturing this wood-based sandwich structure can recover some of the hazardous fuel thinning costs and contribute to acceleration of forest restoration efforts.

## Figures and Tables

**Table 1:** Summary of all experimental specimens subjected to different testing.

Core Type of the Sandwich Panel	Test Type	Number of Specimens	Span Length (*L*) (mm)	Load Span	Width (*b*) (mm)	Thickness (*t*) (mm)
1 layer of corrugated layer	Bending	5	559	*L*/3	203(1-UC)	48
		4	1092	*L*/3	203(1-UC)	48
		4	1219	*L*/3	203(1-UC)	48
		4	2438	*L*/3	203(1-UC)	48
		3	2438	*L*/3	1219(6-UC)	48
	Thermal	7	305	-	305	48
2 layers of corrugated layer	Bending	2	2286	*L*/2	1219(6-UC)	93
	Compression	2	2438	-	1219(6-UC)	93
	Thermal	8	305	-	305	93

**Table 2:** Comparison between bending and compression results of two-layered core sandwich panel with SIP and studded walls. (Coefficient of variation, COV, shown in parentheses).

Test	Specimen	Size (cm) (*L* × *b* × *t*)	# Of Panels	Max. Load (kN)	Equivalent Distributed Load (kPa)	Max. Stress (kPa)
Bending	Sandwich	229 × 122 × 9.3	2	51.1 (Avg. of 53.3 & 49)	44.7	8305
	SIP [41]	244 × 122 × 16.51	3	26.5 (2.47%)	21.7	1458
	Stud [41]	244 × 244 × 16.51	3	68.8 (6.66%)	56.4	4287
Compression	Sandwich	244 × 122 × 9.3	2	181.8 (Avg. of 174.1 & 189.5)	1603	1603
	SIP [41]	274 × 122 × 16.51	3	96.4 (8.39%)	479	479
	Stud [41]	274 × 244 × 16.51	3	250 (21.64%)	621	621

**Table 3:** Comparison of normalized thermal conductivity of one- and two- layered core sandwich panels.

Temperature (°C)			Sandwich Type	Average Thermal Conductivity (*λ*) (*W/mK*)	COV (%)	Diff (%)
Top Plate	Bottom Plate	Mean				
−10	15	2.5	1-layered	0.051	8.23	5.1
			2-layered	0.054	11.23	
0	25	12.5	1-layered	0.052	8.32	3.9
			2-layered	0.054	11.23	
10	35	22.5	1-layered	0.054	8.03	3.7
			2-layered	0.056	11.05	
30	55	42.5	1-layered	0.058	7.34	3.6
			2-layered	0.060	10.42	
50	75	62.5	1-layered	0.063	6.3	2.3
			2-layered	0.065	9.44	

## Data Availability

The raw/processed data required to reproduce these findings cannot be shared at this time due to time limitations, however, data will be provided upon request.
